# Personality, Metabolic Rate and Aerobic Capacity

**DOI:** 10.1371/journal.pone.0054746

**Published:** 2013-01-25

**Authors:** Antonio Terracciano, Jennifer A. Schrack, Angelina R. Sutin, Wayne Chan, Eleanor M. Simonsick, Luigi Ferrucci

**Affiliations:** 1 National Institute on Aging, National Institutes of Health, Department of Health and Human Services, Baltimore, Maryland, United States of America; 2 Florida State University College of Medicine, Tallahassee, Florida, United States of America; University of Sydney, Australia

## Abstract

Personality traits and cardiorespiratory fitness in older adults are reliable predictors of health and longevity. We examined the association between personality traits and energy expenditure at rest (basal metabolic rate) and during normal and maximal sustained walking. Personality traits and oxygen (VO_2_) consumption were assessed in 642 participants from the Baltimore Longitudinal Study of Aging. Results indicate that personality traits were mostly unrelated to resting metabolic rate and energy expenditure at normal walking pace. However, those who scored lower on neuroticism (r =  −0.12) and higher on extraversion (r = 0.11), openness (r = 0.13), and conscientiousness (r = 0.09) had significantly higher energy expenditure at peak walking pace. In addition to greater aerobic capacity, individuals with a more resilient personality profile walked faster and were more efficient in that they required less energy per meter walked. The associations between personality and energy expenditure were not moderated by age or sex, but were in part explained by the proportion of fat mass. In conclusion, differences in personality may matter the most during more challenging activities that require cardiorespiratory fitness. These findings suggest potential pathways that link personality to health outcomes, such as obesity and longevity.

## Introduction

Physical fitness and personality traits are related to lifestyles that promote health [1]–[6] and well-being [7]–[9], predict resilience to diseases and disabilities [1], [10]–[12], such as Alzheimer’s disease [13]–[16], and are robust predictors of longevity [1], [10], [11], [17]–[19]. Physical activity and personality traits are also related. Many studies suggest that those who are more extraverted, conscientious, and less neurotic are more physically active [20]–[22] and those who are more conscientious and less neurotic are less likely to be overweight or obese [5], [23]–[25]. Personality traits have also been related to walking speed and walking speed decline [26], [27]. Little is known, however, about whether personality traits are related to objective measures of energy expenditure and cardiorespiratory fitness. A growing animal literature suggests a link between energetic measures and personality traits such as boldness, activity, exploration, and aggressiveness [28]–[30]. In humans, apart from a few studies with measures of anxiety, hostility, or depression [31], [32], there has not been a systematic investigation of the association between the major dimensions of personality (i.e., neuroticism, extraversion, openness, agreeableness, and conscientiousness) and energy expenditure (volume of oxygen (VO_2_) consumption) at rest and during normal and exertional activity.

Energy expenditure at rest, or resting metabolic rate, reflects the energy required for basal physiological functioning. Energy expenditure during normal-paced walking provides an index of energy required for normal activity, and energy expenditure during peak sustained walking provides an estimate of aerobic capacity that is highly correlated with maximum energetic capacity (VO_2_ max)[33]. Identifying the relationship between personality traits and energy expenditure at these different levels of activity can be informative of the role of psychological processes in energy homeostasis and on individual differences in energetic and aerobic capacity. For example, individuals who are more anxious or aggressive may have a higher resting metabolic rate due to their higher level of activation and reactivity. In contrast, those who are more depressed, introverted or less conscientious might have lower peak aerobic capacity because of their sedentary lifestyle [22]. These traits may shape energy expenditure at rest and during everyday activities, or may only relate to more challenging activities with higher energetic demand. Thus, in this study we examine the association between the five major dimensions of personality, and each of their six facets [34], and energy expenditure at rest and at two levels of exertion. Given the substantial decline of aerobic capacity with age, we further examine whether personality traits show stronger associations with aerobic capacity in the latter part of the lifespan.

## Methods

### Ethics Statement

The Institutional Review Board of the Medstar Research Institute approved the protocol and each participant signed an informed consent form. All clinical investigation has been conducted according to the principles expressed in the Declaration of Helsinki.

### Participants

Participants were part of the Baltimore Longitudinal Study of Aging (BLSA), an ongoing multidisciplinary study of aging implemented by the National Institute on Aging. Concurrent measures of energy expenditure and personality traits were available for up to 642 participants (48% women) with age ranging from 31 to 96 (*M* = 67.01, *SD* = 12.86). Data were collected between July 2007 and November 2011.

### Personality

Personality traits were assessed with the Revised NEO Personality Inventory (NEO-PI-R), a comprehensive measure of the Five Factor Model of personality [34]. The NEO-PI-R consists of 240 items that assesses 6 facets for each dimension of the FFM - neuroticism, extraversion, openness, agreeableness, and conscientiousness. The NEO-PI-R has a robust factor structure that has been replicated in more than 50 cultures [35]. Available longitudinal data over intervals of 10 years indicate that stability coefficients for the five factors are approximately .80 [36].

### Energetics

Energy expenditure (the volume of oxygen consumed) was assessed at rest and at normal and maximal sustained walking speed using a Cosmed k4b^2^ portable metabolic analyzer (Cosmed, Rome, Italy). Prior to testing, the Cosmed was calibrated using a 3.0 liter flow syringe and gases of known concentrations. Oxygen consumption and carbon dioxide production were continuously collected and analyzed with breath-by-breath measurement, and averaged over thirty second intervals to reduce variability. Energy expenditure was calculated as the average volume of oxygen consumed per kilogram of body weight (VO_2_ ml/kg/min) for each test.

#### Resting test

Resting energy expenditure was assessed for 16 minutes in the morning after an overnight stay in a quiet thermo-neutral environment, in a fasted state. To ensure a stable rate of oxygen consumption and reduce variability, the first 5 minutes of values were removed and the average energy expenditure from minutes 5.5–15.5 was used in the analysis.

#### Normal and maximal sustained walking tests

Normal and maximal-sustained walking energy expenditure were assessed during the “long-distance corridor walk” (LDCW), a two-part validated measure of cardiorespiratory fitness [37]. The tests were performed sequentially on a 20-meter corridor course while wearing the Cosmed analyzer. For the first part, participants stood behind a tape starting line and were instructed to walk at their “usual comfortable pace” in a continuous loop until directed to stop. After a command of “Go,” timing was initiated with the first foot-fall over the starting line and stopped after 2.5 minutes. Readings from the first 1.5 minutes of testing were discarded to allow the participant to adjust to the workload and reach stable oxygen consumption and the average of the final minute of testing was used in the analysis. For the second part, participants were escorted back to the starting line and instructed to walk “as fast as possible, at a pace you can sustain for 400 meters.” Timing was initiated with the first foot-fall over the starting line and stopped after 400 meters were completed. Again, to allow the participant to adjust to the workload and reach stable oxygen consumption, the average energy expenditure during maximal sustained walking was derived by removing the first 1.5 minutes of the 400 m walk and averaging the remaining minutes.

### Other measures

Height and weight were assessed by clinical staff using standard methods. A dual-energy X-ray absorptiometry scan was used to assess the proportion of fat and lean tissue. Cigarette smoking was assessed with questions on current and former smoking status.

### Statistical analyses

Partial correlations were used to examine the association between personality traits and energy expenditure measured at rest, normal pace, and peak sustained walking. All analyses controlled for age and sex to account for age and sex differences in both energy expenditure and personality traits [2], [35]. Energy expenditure was expressed in ml per kg of body weight per minute to account for differences in weight. Because walking performance is associated with stride length, models were further adjusted for height. The analyses were repeated using the proportion of fat and lean tissue and cigarette smoking as additional covariates. In multiple regression analyses we tested age and sex interactions with the five broad domains of personality to examine whether the associations differed by age or sex.

## Results

Table 1 presents the characteristics of the study participants and descriptive statistics for energy expenditure at rest and at normal and maximal sustained walking. Participants walked at an average pace of 75 m per minute (average distance: 186 m/time: 2.5 min) during the normal walk task and at an average of 90 m per minute (distance: 400 m/average time: 4.45 min) during the as fast as possible 400 m walk. Energy expenditure at rest was correlated with energy consumed during normal (r = 0.19; p<0.01) and fast walking (r = 0.23; p<0.01), as was energy expenditure between the two walking tasks (r = 0.66; p<0.01). Participants who were older, female, and shorter tended to have lower energy expenditure across the three assessment conditions, especially at the peak walking speed (Table 2).

**Table 1:** Characteristics of study participants.^a^

*Characteristics*	*Value*
Age, mean (SD), years	67.01 (12.86)
Female, No. (%)	310 (48.30)
BMI, mean (SD), Kg/m^2^	27.37 (4.97)
Fat free mass, mean (SD), Kg	48.30 (10.17)
Fat mass, mean (SD), Kg	27.57 (10.74)
Distance at normal walking, mean (SD), m	186.46 (28.75)
Time for 400-m walking corridor, mean (SD), min	4.45 (0.96)
Energy expenditure (VO_2_ ml/kg/min)	
Rest, mean (SD), ml/kg/min	2.64 (0.61)
Normal walking pace, mean (SD), ml/kg/min	12.29 (2.55)
Fast walking pace, mean (SD), ml/kg/min	17.67 (4.78)

Note. N  =  642 for fast walking pace on the 400-m walking corridor, N  =  634 for normal walking, N  =  441 for rest data.

**Table 2:** Partial correlations of personality traits, energy expenditure (volume of oxygen (VO_2_) consumption), and related measures at rest, normal, and fast walking tasks.^a^

	Rest	Normal walking		Fast walking	
	VO_2_	Distance	VO_2_	Time	VO_2_
Age	-.14**	-.41**	-.05	.55**	-39**
Sex	-.11*	-.08	-.14**	.14**	-.19**
Height	.10*	.22**	.14**	-.30**	.27**
Neuroticism	.00	-.08	-.02	.14**	-.12**
Extraversion	-.01	.08*	.05	-.13**	.11**
Openness	.04	.08*	.09*	-.13**	.13**
Agreeableness	-.07	-.02	-.05	-.03	-.01
Conscientiousness	.00	.09*	.03	-.09*	.09*
N1: Anxiety	-.06	-.05	-.01	.09*	-.08*
N2: Angry Hostility	.08	-.01	.03	.11**	-.07
N3: Depression	-.01	-.09*	-.01	.14**	-.10*
N4: Self-consciousness	-.06	-.04	-.04	.05	-.08*
N5: Impulsiveness	.07	-.08*	-.01	.11**	-.10*
N6: Vulnerability	-.02	-.07	-.03	.10*	-.10*
E1: Warmth	-.06	.06	.01	-.07	.05
E2: Gregariousness	-.04	.01	-.04	-.04	.00
E3: Assertiveness	.04	.08	.08*	-.08*	.10*
E4: Activity	.06	.20**	.18**	-.24**	.21**
E5: Excitement-Seeking	.00	-.05	-.04	.00	.02
E6: Positive Emotions	-.05	.07	.05	-.13**	.08*
O1: Fantasy	.01	.04	.03	-.03	.04
O2: Aesthetics	.03	.05	.05	-.04	.03
O3: Feelings	.06	.03	.05	-.05	.09*
O4: Actions	-.01	.11**	.09*	-.16**	.13**
O5: Ideas	-.02	.03	.07	-.08	.12**
O6: Values	.09	.07	.07	-.17**	.14**
A1: Trust	-.03	.07	.07	-.09*	.11**
A2: Straightforwardness	-.06	-.02	-.04	-.02	.01
A3: Altruism	-.04	.03	.00	-.09*	.04
A4: Compliance	-.10*	-.08*	-.08*	.00	-.01
A5: Modesty	-.06	-.02	-.07	.05	-.10*
A6: Tender-mindedness	.01	-.04	-.07	.03	-.09*
C1: Competence	-.03	.05	.00	-.04	.02
C2: Order	.01	.06	.03	-.07	.05
C3: Dutifulness	.02	.06	.04	-.09*	.11**
C4: Achievement Striving	.06	.09*	.07	-.09*	.13**
C5: Self-Discipline	.03	.10*	.05	-.11**	.06
C6: Deliberation	-.08	.04	-.05	-.01	.02

Note. N = 642 for peak walking speed on the 400-m walking corridor, N = 634 for normal walking, N = 441 for rest data. Covariates: age, sex, and height. * p<0.05; **p<0.01

Personality traits were largely unrelated to resting energy expenditure (Table 2). Energy expenditure during normal walking was associated with higher scores on the openness factor and with 4 of the 30 facets (ps < 0.05). In contrast, 4 of the 5 broad domains and 17 of the 30 facets were associated with energy expenditure at maximal sustained walking speed. Specifically, those who scored lower on neuroticism and higher on extraversion, openness, and conscientiousness had higher peak energy expenditure (ps<0.05). Using Meng et al. [38] formula to compare correlated correlation coefficients, the associations of neuroticism, extraversion, openness, and conscientiousness with peak energy expenditure were significantly different from those with resting metabolic rate (ps<.05; one-tail). In supplementary analyses that accounted for age and sex, those who scored high on neuroticism (top 25%), or low on extraversion, openness, and conscientiousness (bottom 25%) consumed between 1 and 2 ml O_2_/kg/min less than those at the other end of the distribution, as if they were roughly 10 years older.

We repeated the analyses accounting for differences in the proportion of lean and fat mass, and found similar results to those reported in Table 2. At the factor level, however, the association between openness and normal walking energy expenditure was reduced to a trend (r = 0.07; p = 0.08), and the association of conscientiousness with maximal sustained walking energy expenditure was reduced to non-significance (r = 0.06; p = 0.17). The associations were unchanged when adjusting for smoking status. We further examined whether the associations between personality traits and energy expenditure were dependent on age or sex. Out of 30 interactions tested (5 traits by age and sex for each of the three levels of energy expenditure), we found only one significant interaction: the association between conscientiousness with maximal sustained walking energy expenditure was stronger for men (βc_*_sex = −0.07, p = 0.04).

As reported elsewhere [33], performance on the 400 m walk component of the LDCW is strongly associated with aerobic capacity. Thus, it was not surprising that the correlations between personality and walking speed were similar to those between personality and energy expenditure (see Table 2). We also examined “energetic efficiency” during maximal sustained walking by computing energy expenditure per meter walked (peak_VO_2 (ml/kg/min)_/time_400 m). Participants who scored lower on neuroticism (r = −0.09; p<.05), and higher on extraversion (r = 0.10; p<.05), openness (r = 0.13; p<.05), and conscientiousness (r = 0.08; p<.05) had higher efficiency, that is they required less energy to complete the 400 m walk.

## Discussion

In a sample that spanned from middle adulthood to old age, we found that individuals low in neuroticism and high in extraversion, openness, and conscientiousness demonstrated higher aerobic capacity at maximal sustained walking speed. Personality, however, was mostly unrelated to metabolic rate at rest and during normal walking. These results indicate that personality differences emerge mostly when greater energetic effort is required, with a resilient personality profile associated with higher aerobic capacity. This observation is consistent with evidence linking these same personality traits, particularly low neuroticism and high conscientiousness, to better health and longevity [4], [5], [17]–[19].

The associations between personality and aerobic capacity at maximal sustained walking speed could be due to a number of mechanisms. Individuals with a more resilient personality profile (e.g., low neuroticism and high conscientiousness) are more likely to maintain an active lifestyle [22], are less likely to be overweight or obese [5], [24], and are less likely to engage in other health risk behaviors, such as cigarette smoking [39]. Because of their healthy lifestyle, and through more direct physiological pathways, individuals low in neuroticism and high in conscientiousness are less likely to have metabolic syndrome [40], have lower levels of inflammatory markers [41], and lower illness burden [12], [42]. Aerobic capacity may be one mechanism through which personality traits contribute to better health and longevity. Likewise, greater aerobic capacity might be one factor that shapes personality, especially for traits that are expressed through behaviors that require a higher level of energy, such as extraversion.

In addition to behavioral and physiological mechanisms, personality traits might be linked to peak performance through more “motivational” psychological processes. Some individuals simply do not put as much effort in the task as others; some might push themselves to their limit while others do not. For example, those who score lower on dutifulness are less committed and less likely to strictly adhere to task instructions, and those who score lower on achievement striving are more lackadaisical and less driven [34]. Fear of falling, lower tolerance for pain, or fatigue may also slow down some of the subjects, perhaps those scoring lower on extraversion, openness, conscientiousness, and higher on neuroticism. Such individuals might exhibit lower peak performance because they did not follow instructions and did not walk as fast as they could. Although these motivational and emotional processes may play a role in how fast a person chooses to walk and to some extent on how much oxygen they consume, we found essentially the same associations between the five major dimensions of personality and energy expenditure after accounting for differences in walking speed. In other words, those with a more resilient personality profile were not just faster and with greater aerobic capacity, but they were also more efficient in their energy expenditure while walking. That is, they could go faster with relatively less energy. This better energetic efficiency is unlikely to be explained by the above mentioned differences in motivation.

The activity facet of extraversion had the strongest association with walking energy expenditure. Active individuals tend to be energetic, keep busy, and live an active and fast-paced life. Thus, it is not surprising that those who are dispositionally active would have greater aerobic capacity. Individual differences in the level of activity is a temperamental trait that emerges early in life, and activity is perhaps the personality trait that has the steepest decline in old age [43], paralleling the physiological decline in energy availability. In addition to activity, two other facets of extraversion, assertiveness and positive emotions, were also associated with higher aerobic capacity. Assertive individuals tend to be forceful and dominant, and may be more likely to take the lead in part because of a higher energy level [30]. A large literature links well-being to health outcomes, with many studies [44]–[46], but not all [47], finding greater emotional well-being associated with reduced risk of cardiovascular diseases and mortality. The current study suggests that aerobic capacity might be a potential mediator of the links between positive emotions and cardiovascular health.

We found lower aerobic capacity to be associated with most facets of neuroticism, a domain strongly associated with risk of depression and other mental health conditions [48]. The association of neuroticism with aerobic capacity is consistent with evidence linking exercise with fewer anxiety and depressive symptoms, but the causal relation between depressive symptoms and physical activity remains unclear [21]. Indeed, among older adults, fear of falling and higher anxiety impair performance in balance tasks, reduce stride length and slow walking gait [49]–[51].

The strong associations between aerobic capacity and the openness domain were relatively unexpected, although some evidence are consistent with such association [26], [52]–[54]. For example, a number of studies have shown that those higher on openness report better dietary quality [55], [56], and better eating habits may be one factor that contributes to better physical fitness. Within the openness domain, the most consistent effects were seen for the facet openness to action, which assesses a willingness to engage in different activities, to go and explore new places. Such preference for novel activities may promote greater physical activity, which may explain the observed associations. The better aerobic capacity of those high on openness to actions is consistent, and may partly explain, the finding that high openness to actions is prospectively associated with decreased cardiac mortality and all-cause mortality among patients with coronary artery disease [57].

Of the five domains of personality, we found no association with agreeableness. This is somewhat surprising given that antagonistic individuals are likely to engage in health risk behaviors, such as cigarette smoking [39] and they tend to have thicker arteries [58] and are at greater risk of cardiovascular diseases [59].

The associations between personality traits and energy expenditure did not vary by age or by sex. Both resting and maximal energy expenditure decline with age, albeit at different rates. We found, however, that personality traits shared similar associations with energy expenditure regardless of age. These cross-sectional analyses suggest that the association between personality and energy expenditure does not differ markedly across the middle to latter part of the lifespan. The pattern of associations was also similar for men and women. Most of the associations remained significant even after accounting for body composition parameters and cigarette smoking. Conscientiousness was the exception, with part of this association accounted for by the proportion of lean and fat tissue. Individuals that score low on conscientiousness may have lower aerobic capacity because of the higher proportion of fat tissue. The association between personality, physical fitness and obesity is likely to be complex, with reciprocal influences. Future studies could test to what extent physical fitness mediates the association between personality and obesity. Interestingly, our findings suggest that the personality-obesity association (and similarly the personality links to health and longevity) is more likely to be mediated by peak energetic capacity rather than resting metabolic rate.

Among the limitations of this study is the selective nature of the sample. BLSA participants tend to be more educated and from higher socio-economic status compared to the general population. Given that multiple tests were performed, there is an increased likelihood of false positive results. Before reaching firm conclusions, these findings need to be replicated in independent samples. Another limitation is the cross-sectional nature of the analyses. Only a few participants in our sample had multiple assessments of energy expenditure using the same procedure, but it would be interesting to examine whether personality traits predict the differential rate of decline in aerobic capacity with aging. It is also likely that aerobic capacity and health status influence changes in personality over the lifespan. Lower energy level might be associated with more pronounced changes in personality, with accelerated changes toward the personality profile typical of older adults (e.g., less open, less extroverted)[35], [43].

In conclusion, the reported associations highlight the links between personality traits and cardiorespiratory fitness in older adults, both of which are powerful predictors of disability and mortality. We found a strikingly contrasting pattern, with personality traits related to aerobic capacity at peak walking speed, but unrelated to resting metabolic rate. In addition to the links between personality traits and energy expenditure, this study is informative on the role of psychological traits in lifestyles associated with successful aging.
